# Diversity and Co-occurrence Patterns of Soil Bacterial and Fungal Communities in Seven Intercropping Systems

**DOI:** 10.3389/fmicb.2018.01521

**Published:** 2018-07-06

**Authors:** Sen Li, Fengzhi Wu

**Affiliations:** ^1^Department of Horticulture and Landscape Architecture, Northeast Agricultural University, Harbin, China; ^2^Heilongjiang Province Key University Laboratory of Cold Area Vegetable Biology, Northeast Agriculture University, Harbin, China; ^3^Ministry of Agriculture Key Laboratory of Biology and Germplasm Enhancement of Horticulture crops in Northeast China, Northeast Agricultural University, Harbin, China

**Keywords:** soil microbe, intercropping, bacteria, fungi, co-occurrence networks

## Abstract

Intercropping plays a vital role in greenhouse production, and affects soil physicochemical properties and soil microbial communities structure, but influences of intercropping on the relationship of microorganisms are reported in continuous cropping soil rarely. Here, we investigated the effects of seven intercropping systems [alfalfa (*Medicago sativa* L.)/cucumber, trifolium (*Trifolium repens* L.)/cucumber, wheat (*Triticum aestivum* L.)/cucumber, rye (*Secale cereale* L.)/cucumber, chrysanthemum (*Chrysanthemum coronrium* L.)/cucumber, rape (*Brassica campestris* L.)/cucumber, mustard (*Brassica juncea* L.)/cucumber] on soil bacterial and fungal communities compared to the cucumber continuous cropping system in the greenhouse. The results showed that intercropping increased microbial OTU richness and fungal communities diversity, soil bacterial communities diversity was abundant in the trifolium-cucumber and mustard-cucumber systems. Nevertheless, there was no significant differences of microbial communities structure between intercropping and monoculture systems. Redundancy analysis indicated that soil microbial communities composition was indirectly influenced by soil properties. In addition, network analysis demonstrated that simple inter-relationships of fungal taxa were observed in the intercropping soil, and trifolium, wheat, and mustard intercropping systems had a complex connection between bacterial taxa. Taken together, trifolium and mustard as the intercrops significantly increased cucumber continuous cropping soil bacterial and fungal communities diversity. Moreover, intercropping strongly changed the relationships of microbial taxa, though did not shape notably soil microbial communities structure.

## Introduction

Agricultural management practices and cropping systems can evidently influence crop yield, soil physicochemical characteristics, and soil microbial activity and composition, and they have attracted attention in agricultural production so far (Acosta-Martínez et al., 2010; Li et al., 2010; Singh et al., 2016). Intercropping, the coinstantaneous cultivation of more than one crop species in the same location, is attributed to the efficiency and complementation of resources in temporal and spatial patterns, and to the enhancement of the resistance to diseases, pests, and hostile plants (Li et al., 1999; Banik et al., 2006; Hinsinger et al., 2011; Brooker et al., 2015). However, not all of the intercropping systems can tend to the better. Evidence illustrated that legume and cereal species intercropped had lower biomass and nitrate accumulation than sole crop (Li et al., 2001; Corre-Hellou et al., 2011). Understanding the roles of several intercropping systems can be conducive to explore new strategies to improve agricultural development in a sustainable way.

Soil microorganisms play an important role in soil biogeochemical processes such as nitrogen, phosphorus and other elements cycles (de Graaff et al., 2010; Mangan et al., 2010; Urbanová et al., 2015). Soil microbial communities composition and diversity are imperative to maintain the plant biodiversity, soil health and productivity (van der Heijden et al., 1998; Mangan et al., 2010). Furthermore, several reports showed that changes in soil microbial diversity and structure are confirmed to be connected with plant species, soil physicochemical characteristics as well as land-use types (Lauber et al., 2008; Mitchell et al., 2010; Bell et al., 2013). However, little is known about the influence of intercropping on continuous cropping soil microbial communities.

Soil microbes were not isolated in the microbial community, but existed in the complicated interaction systems that decided microbial community structure (Freilich et al., 2010). In previous study, most analytical methods of soil microbial communities were applied to illustrate microbial diversity, community composition and their variations with biotic or abiotic factors, but were rarely able to explain the relationships between soil microbial species (Deng et al., 2012). Co-occurrence network analysis gives new insight into the inter-species interactions of soil microbial communities, and promotes the understanding of the niche spaces among community members (Barberán et al., 2012). Nevertheless, the microbial interactions of intercropping systems were poorly understood in continuous cropping soil.

In the present study, we performed a metagenomic analysis of bacterial and fungal communities based on Miseq sequencing of 16S rRNA and ITS genes to illustrate their variation in the seven intercropping systems. The purpose of this study was to explore the responses of microbial communities to intercropping systems on the condition of continuous cropping soils. We hypothesized that intercropping influenced soil bacterial and fungal communities by variation of soil properties, and co-occurrence patterns of bacterial and fungal taxa distinctly responded to several intercropping systems in the continuous cropping soils.

## Materials and methods

### Field site description and experimental design

The experiment was located at Horticultural Experimental Station Northeast Agricultural University (45°41′N, 126°37′E), Harbin, Heilongjiang Province, China. The field soil was black soil (Mollisoil), that was under continuous cucumber cropping for 3 years. The soil chemical properties, measured by Bao (2005), were as follows: pH 6.64; EC, 0.88 mS cm^−1^; ${\text{NH}}_{4}^{+}$-N 13.32 mg kg^−1^; ${\text{NO}}_{3}^{+}$-N, 253.04 mg kg^−1^; available phosphorus (AP), 277.62 mg kg^−1^; available potassium (AK), 359.03 mg kg^−1^.

The experiment was a randomized block design with three replicates in the greenhouse, and the size of each plot was 6 × 0.5 m. 7 crops [alfalfa (A), trifolium (T), wheat (W), rye (Ry), Chrysanthemum (C), rape (Ra), and mustard (M)] were intercropped with cucumber as experimental group, and cucumber monoculture (CM) was the control. The spring experiment were ranged from 21 April 2015, cucumber seedlings 3-true-leaves, to 30 June 2015, cucumber harvest period. 12 cucumber plants were in one row as the test district, and two protective lines were on both sides. After 10 days of planting the cucumber, the seeds of the intercrops were sowed on the outside of cucumber about 10 cm. The number of intercrop seeds was as follows: (I) wheat and rye were 30 seedlings, respectively; (II) trifolium and alfalfa were 40 seedlings, respectively; (III) rape and mustard were 3 seedlings, respectively; (IV) chrysanthemum was 5 seedlings. When the intercrops grew to 20 cm high, it was pruned for 10 cm to avoid effects on growth of cucumber and their residue were left on soil surface. The compound NPK fertilizer (16:16:8) was performed with 300 kg ha^−1^ for each plot. The fall trial began on 29 July and ended on 6 October, and the specific operation was consistent with that in spring. A more detailed description of the experiment can be referred to (Chang et al., 2017).

### Soil sampling and soil physicochemical properties analysis

The soil samples, totally 48, were obtained from 24 test district on 30 June and 6 October, and then were mixed and sieved through a 2 mm mesh to thoroughly homogenize and remove the roots, plant residues and stones. Afterwards the soils were transferred to the laboratory, every sample was divided into two parts: some archived at −80°C for DNA extraction, and the others for chemical analyze stored at −4°C.

Soil physicochemical characteristics were analyzed based on Bao (2005). Soil pH and EC were determined in a soil water suspension (1:2.5 w/v) using a glass electrode and a conductivity meter, respectively. For soil inorganic nitrogen concentration (${\text{NH}}_{4}^{+}$-N, ${\text{NO}}_{3}^{-}$-N), available P and K were extracted with 2 M KCl, 0.5 M NH4^+^OAc (pH = 7) and 1 M NaHCO_3_ (pH = 8.5), respectively, and then soil filtrates were determined by Continuous Flow Analyser (SAN++, Skalar, Breda, Netherlands). Soil moisture contents were determined by drying at 105°C for 24 h.

### DNA extraction, PCR and Miseq sequencing

Soil total DNA was extracted from 0.5 g soil using the MoBioPowerSoil™ DNA Isolation Kit (Mo Bio Laboratories Inc. Carlsbad, CA, USA) according to the manufacturer's instructions. DNA concentration and purity were determined with a NanoDrop 2000 Spectrophotometer (Thermo Scientific, USA).

The V3-V4 hypervariable region of the 16S rRNA gene and the ITS1 region of fungal ITS gene were used as the bacterial-specific fragment and the fungal-specific fragment with the 338F/806R (Xu et al., 2016) and ITS1F/ITS2 (Bellemain et al., 2010) primer sets for bacteria and fungi, respectively. These primer pairs were modified with a 6-bp unique barcode sequence at the 5′ end to identify samples. All amplification was performed in 25 μl reactions contained 0.5 μl of each primer, 1 μl of template, 2 μl 2.5 mM dNTPs, 0.5 μl of FastPfu Polymerase (Transgen Biotech, Beijing, China), 0.5 μl of × 5 FastPfu buffer, and 20 μl deionized H_2_O. The PCR conditions, performed in an ABI GeneAmp® 9700 PCR System (ABI, MA, USA), were described as follows: 5 min of initial denaturation step at 94°C, followed by 35 cycles of 94°C for 30 s, 50°C for 30 s and 72°C for 30 s, and a final extension step at 72°C for 10 min for the 16S V3-V4 rRNA gene; and 3 min of initial denaturation step at 95°C, followed by 35 cycles of 94°C at 30 s, 55°C for 30 s and 72°C for 45 s, and a final extension at 72°C for 10 min for ITS genes. The products from the three replicate amplifications of the bacterial 16S rRNA gene and fungal ITS gene were separately pooled and evaluated on 2% agarose gels (TBE buffer). Amplicons were purified with a DNA gel extraction kit (Axygen, China), quantified with a QuantiFluorTM-ST fluorometer (Promega, Madison, WI, USA), pooled at equimolar concentrations, and finally sequenced on an Illumina Miseq PE300 platform at Majorbio Bio-Pharm Technology Co., Ltd. (Shanghai, China).

### Soil bacterial and fungal abundance analysis

Quantification of 16S rRNA and ITS genes were performed on a iQ5 Real-Time PCR Detection System (Bio-Rad, USA) with primers 338F/518R (Muyzer et al., 1993) and ITS1F/ITS4 (Gardes and Bruns, 1993), respectively. The PCR reaction mix contained 10 μl SYBR Green I PCR master mix (Applied Biosystems, USA), 0.2 μl each primer (10 μ mol l^−1^), 2.5 μl template DNA (sample DNA or plasmid DNA for standard curves), and finally fit it up with sterile deionized water to 20 μl. The qPCR conditions contained an initial denaturation at 95°C for 5 min, followed by 37 cycles of denaturation at 95°C for 45 s, annealing at 56°C or 58°C for 45 s for bacteria and fungi, respectively. The standard samples were diluted to yield a series of 10-fold concentrations and subsequently used for qPCR standard curves, The *R*^2^-value for each standard curve exceeded 0.99, indicating linear relationships over the concentration ranges used in this study. All of the amplifications were run in triplicate with the DNA extracted from each soil sample.

### Sequence data analysis

The raw data yielded from Illumina sequencing were analyzed using QIIME software (v1.9.0) and the UPARSE pipeline as described before (Zhong et al., 2015). The UPARSE pipeline was performed for taxonomic assignment with similarities >97% (Edgar, 2013). Taxonomic classification was conducted with SILVA (version 119; http://www.arb-silva.de) and UNITE (version 7.0; http://unite.ut.ee/index.php) databases for bacteria and fungi, respectively. To preclude bias as a result of several sequencing depth, all samples were subsequently subsampled based on the minimum number of soil microbial sequencing depth of this study. The raw data have been deposited in the NCBI SRA database (SRP122874).

### Statistical analysis

The bacterial and fungal diversity indices, Chao 1 richness, Shannon index, Simpson index and coverage were calculated by QIIME (Caporaso et al., 2010). Heatmap analysis was used to compare the top 50 classified genera in per sample of two growing seasons with the gplot package in R (R v.3.2.5) (R Development Core Team, 2006). Non-metric multidimensional scaling (NMDS) and Redundancy analysis (RDA) were carried out to reveal the microbial structure and the relationship between environmental factors and microbial abundance, and Anosim (analysis of similarity), adonis (non-parametric MANOVA), and MRPP (multi-response permutation procedure) were used to compare the microbial community differences of two cropping seasons (24 samples per season) with the Bray-Curtis distance and 999 permutations, they were performed in R using the vegan package. To demonstrate the relationship of different species among several samples, network analysis based on spearman's rank analysis in this study was performed using the 50 most abundant genera of bacterial and fungal communities. The co-occurrence patterns of soil microbial communities were explored based on strong (ρ > 0.6) and significant correlations (P < 0.01), and were visualized with the Gephi (Jacomy et al., 2009). The size of each node represented the number of connections, the node was colored by taxonomy. Soil properties, bacterial and fungal abundances, alpha diversity indices, and spearman's rank analysis were performed at 0.05 probability level in SPSS software (Version 17.0).

## Results

### Soil physicochemical properties

Soil physicochemical properties of all samples were summarized in Table 1. Compared with cucumber monoculture, soil AP content was significantly (*P* < 0.05) decreased under intercropping systems in the spring. rye-cucumber system had a significantly (*P* < 0.05) higher soil ${\text{NH}}_{4}^{+}$-N content and ${\text{NO}}_{3}^{-}$-N content in the spring, and alfalfa-cucumber and chrysanthemum-cucumber systems significantly (*P* < 0.05) increased soil ${\text{NH}}_{4}^{+}$-N content and ${\text{NO}}_{3}^{-}$-N content in the fall. Wheat-cucumber and rye-cucumber systems had a significantly (*P* < 0.05) higher EC in the two cropping seasons. No significant differences were found in soil moisture content, soil pH and AK content between intercropping and monoculture systems.

**Table 1:** Effects of different crop modes on soil chemical properties at two sampling times in a black soil.

**Crop season^a^**	**Treatments^b^**	**Moisture (%)**	**pH**	**EC (mS·cm^−1^)**	**${\text{NH}}_{4}^{+}$-N (mg·kg^−1^)**	**${\text{NO}}_{3}^{-}$-N (mg·kg^−1^)**	**AP^c^ (mg·kg^−1^)**	**AK^c^ (mg·kg^−1^)**
S	A	24.94 ± 5.44a	7.32 ± 0.05a	0.14 ± 0.01cd	27.66 ± 8.17b	35.13 ± 9.37a	467.14 ± 53.44b	533.20 ± 110.66a
	T	23.44 ± 0.41a	7.18 ± 0.05a	0.12 ± 0.00d	15.33 ± 6.96b	63.61 ± 26.32a	427.53 ± 51.22b	561.11 ± 78.41a
	W	22.47 ± 0.71a	7.16 ± 0.09a	0.31 ± 0.02a	16.51 ± 2.76b	62.78 ± 24.81a	308.54 ± 83.92c	566.91 ± 49.71a
	Ry	22.54 ± 4.01a	7.22 ± 0.15a	0.25 ± 0.05b	56.31 ± 28.18a	54.58 ± 25.28a	276.72 ± 66.06c	460.27 ± 21.98a
	C	19.51 ± 1.40a	7.22 ± 0.17a	0.15 ± 0.04cd	30.37 ± 11.53b	41.65 ± 14.32a	237.93 ± 49.81c	589.08 ± 49.52a
	Ra	21.00 ± 4.73a	7.17 ± 0.09a	0.19 ± 0.00c	20.39 ± 9.53b	37.18 ± 15.57a	430.65 ± 50.62b	619.80 ± 160.82a
	M	24.71 ± 2.66a	7.20 ± 0.21a	0.15 ± 0.00cd	35.92 ± 6.91ab	33.29 ± 14.66a	505.27 ± 111.20b	503.53 ± 54.84a
	CM	23.41 ± 1.31a	7.10 ± 0.06a	0.13 ± 0.01cd	26.77 ± 9.94b	44.96 ± 21.57a	621.85 ± 41.44a	617.54 ± 36.36a
F	A	25.50 ± 2.86a	6.81 ± 0.14a	0.17 ± 0.01b	13.15 ± 3.86a	43.64 ± 2.99a	388.36 ± 40.70a	546.25 ± 37.31a
	T	26.68 ± 1.36a	6.78 ± 0.05a	0.22 ± 0.04b	12.82 ± 2.92a	36.51 ± 3.58ab	386.53 ± 37.81a	517.75 ± 26.07b
	W	19.98 ± 8.27a	6.91 ± 0.05a	0.37 ± 0.07a	22.25 ± 8.56a	34.14 ± 3.03b	390.63 ± 19.06a	431.84 ± 38.31b
	Ry	26.49 ± 2.73a	6.85 ± 0.05a	0.42 ± 0.05a	17.63 ± 3.03a	34.25 ± 7.82b	427.94 ± 12.86a	530.36 ± 70.57ab
	C	25.82 ± 1.76a	6.80 ± 0.06a	0.38 ± 0.04a	19.60 ± 7.38a	43.91 ± 3.41a	358.98 ± 26.13a	525.23 ± 56.65ab
	Ra	24.39 ± 0.88a	6.84 ± 0.08a	0.23 ± 0.02b	19.36 ± 8.49a	39.16 ± 8.27ab	368.92 ± 59.10a	506.17 ± 95.25ab
	M	25.87 ± 2.33a	6.86 ± 0.11a	0.19 ± 0.01b	21.33 ± 3.87a	32.78 ± 1.70b	393.76 ± 34.35a	515.80 ± 40.82ab
	CM	19.11 ± 9.03a	6.75 ± 0.08a	0.26 ± 0.06b	18.81 ± 4.15a	33.66 ± 1.72b	425.29 ± 30.81a	521.20 ± 22.51ab

a *S and F indicated experiment conducted in spring and fall seasons, respectively*.

b *A, Alfalfa; T, Trifolium; W, Wheat; Ry, Rye; C, Chrysanthemum; Ra, Rape; M, Mustard; CM, Cucumber monoculture*.

c *AP, AK indicated soil available phosphorus and available potassium, respectively*.

*Data with different letters in each column indicate significantly different between treatments at 0.05 level*.

### Soil bacterial and fungal abundance

**Figure 1:**
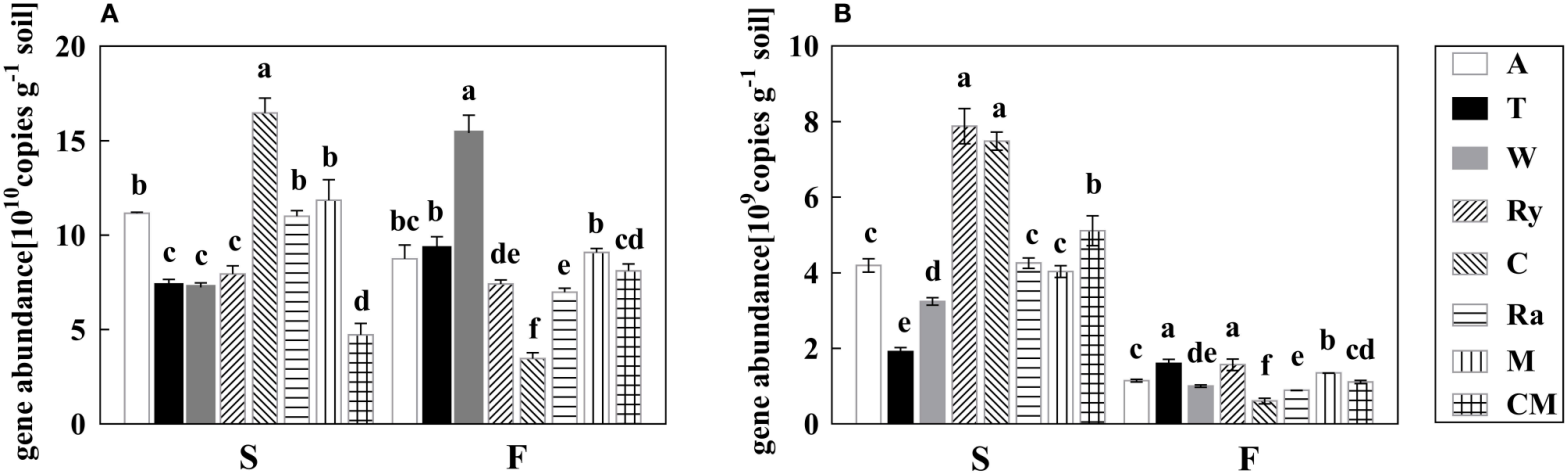
The abundances of bacterial 16S rRNA gene **(A)** and fungal ITS gene **(B)** under different treatments in the spring (S) and fall (F) cropping seasons. Data with different letters in each column indicate significantly different between treatments at 0.05 level.

Soil bacterial abundance was significantly (*P* < 0.05) higher under all intercropping systems than cucumber monoculture in the spring, and wheat-cucumber, trifolium-cucumber, mustard-cucumber systems significantly (*P* < 0.05) increased bacterial abundance in the fall (Figure 1A). Besides, chrysanthemums-cucumber and rape-cucumber systems had significantly lower bacterial abundance in the fall (*P* < 0.05), and the highest bacterial abundance was existed in chrysanthemum-cucumber and wheat-cucumber systems in the two growing seasons, respectively.

Compared with cucumber monoculture, fungal abundance significantly (*P* < 0.05) decreased under intercropping systems except rye-cucumber and chrysanthemum-cucumber systems in the spring, and chrysanthemum-cucumber and rape-cucumber systems had a significantly (*P* < 0.05) lower fungal abundance in the fall (Figure 1B). Rye-cucumber and chrysanthemum-cucumber systems in the spring, and trifolium-cucumber, rye-cucumber, and mustard-cucumber systems in fall were significantly (*P* < 0.05) increased fungal abundance.

### Soil microbial community diversity and composition

The process of raw sequencing reads generated about the total of 1788705 high quality V3-V4 sequences and 1798779 high quality ITS1 sequences, average read length of bacteria and fungi were 437 and 262 bp, and sequences of all samples were clustered to 134463 bacterial OTUs and 15541 fungal OTUs with a 97% identity threshold, respectively. The alpha-diversity indices of bacterial and fungal communities were observed for Table 2. Bacterial and fungal diversity represented by Shannon index and Simpson index. Bacterial Shannon index of trifolium-cucumber and mustard-cucumber systems were remarkably higher than monoculture in spring and fall (*P* < 0.05), However, for fungal Shannon index, every treatment was significantly higher than monoculture under two growing seasons except mustard-cucumber system in the spring. Simpson index of bacterial and fungal communities were opposite to Shannon index. Bacterial richness, estimated by Chao1 index, was higher under rape-cucumber, wheat-cucumber, and rye-cucumber systems than under monoculture in spring, and under intercropping systems except wheat-cucumber and chrysanthemum-cucumber systems in fall. For fungal richness, intercropping systems were significantly higher than cucumber monoculture in spring, but mustard-cucumber systems were significantly lower than monoculture in fall (*P* < 0.05). The coverage of soil bacterial and fungal communities was more than 95 and 99%, showing that the current sequencing depth in this study was enough to cover the soil bacterial and fungal communities diversity, respectively.

**Table 2:** Diversity indices of soil microbial communities based on 16S rRNA and ITS genes analyzed from Illumina Miseq sequencing.

**Classified**	**Crop season**	**Treatments**	**Sequences**	**Number of OTUs**	**Shannon**	**Simpson**	**Chao 1**	**Coverage (%)**
Bacteria	S	A	39, 665 ± 766	2, 857 ± 32cd	6.69 ± 0.02de	0.0053 ± 0.0003a	3871.88 ± 10cd	95.58
		T	36, 371 ± 173	2, 977 ± 63ab	6.80 ± 0.03c	0.0042 ± 0.0003b	3832.83 ± 39d	95.67
		W	39, 824 ± 3, 593	2, 934 ± 23ab	6.82 ± 0.01bc	0.0037 ± 0.0002c	4112.14 ± 5ab	95.44
		Ry	40, 889 ± 1, 004	2, 954 ± 19ab	6.89 ± 0.01a	0.0031 ± 0.0000d	4118.91 ± 12ab	95.55
		C	43, 255 ± 1, 576	2, 824 ± 68de	6.82 ± 0.03bc	0.0032 ± 0.0001d	3887.53 ± 37c	95.59
		Ra	40, 746 ± 810	2, 985 ± 6a	6.83 ± 0.01b	0.0034 ± 0.0004cd	4158.64 ± 47a	95.41
		M	31, 087 ± 104	2, 909 ± 31bc	6.72 ± 0.01d	0.0054 ± 0.0001a	3858.57 ± 27cd	95.62
		CM	32, 749 ± 1, 751	2, 763 ± 13e	6.68 ± 0.01e	0.0055 ± 0.0001a	4100.61 ± 20b	95.56
	F	A	32, 187 ± 841	2, 855 ± 9a	6.62 ± 0.05b	0.0060 ± 0.0000c	3877.42 ± 12b	95.56
		T	42, 883 ± 59	2, 754 ± 70a	6.85 ± 0.13a	0.0041 ± 0.0000e	4035.48 ± 55a	95.62
		W	39, 120 ± 334	2, 623 ± 112b	6.53 ± 0.02bc	0.0073 ± 0.0002a	3313.75 ± 64e	96.12
		Ry	31, 186 ± 700	2, 650 ± 26b	6.55 ± 0.02bc	0.0054 ± 0.0003d	3683.83 ± 65c	96.01
		C	32, 438 ± 917	2, 621 ± 63b	6.60 ± 0.11bc	0.0056 ± 0.0002d	3587.66 ± 2d	96.12
		Ra	40, 137 ± 1, 565	2, 783 ± 61a	6.60 ± 0.02bc	0.0066 ± 0.0002b	4002.13 ± 16a	95.54
		M	37376 ± 310	2, 850 ± 21a	6.75 ± 0.02a	0.0037 ± 0.0001f	3829.88 ± 18b	95.65
		CM	41, 353 ± 657	2, 582 ± 10b	6.48 ± 0.06c	0.0075 ± 0.0001a	3637.16 ± 29cd	95.90
Fungi	S	A	39, 257 ± 1, 609	323 ± 5ab	3.36 ± 0.21a	0.0761 ± 0.0005cd	439.86 ± 1b	99.68
		T	33, 031 ± 122	313 ± 5bc	3.34 ± 0.06a	0.0656 ± 0.0023d	393.95 ± 6d	99.68
		W	35, 871 ± 2, 329	330 ± 9a	3.47 ± 0.16a	0.0667 ± 0.0113d	464.74 ± 14a	99.61
		Ry	31, 575 ± 1, 165	302 ± 8c	3.40 ± 0.09a	0.0641 ± 0.0116d	417.72 ± 11c	99.71
		C	41, 051 ± 2, 684	285 ± 3d	3.34 ± 0.05a	0.0674 ± 0.0081d	376.85 ± 8d	99.72
		Ra	41, 839 ± 687	313 ± 5bc	3.39 ± 0.12a	0.0908 ± 0.0155bc	424.45 ± 8bc	99.70
		M	38, 081 ± 1, 727	268 ± 4e	3.06 ± 0.05b	0.1018 ± 0.0026ab	349.53 ± 11e	99.74
		CM	32, 256 ± 1, 321	254 ± 13f	2.93 ± 0.06b	0.1113 ± 0.0073a	329.54 ± 20f	99.71
	F	A	32, 917 ± 1, 138	358 ± 3b	3.62 ± 0.04a	0.0567 ± 0.0097cd	481.98 ± 3a	99.63
		T	37, 713 ± 731	387 ± 7a	3.70 ± 0.07a	0.0538 ± 0.0068d	501.87 ± 20a	99.64
		W	31, 899 ± 393	350 ± 4b	3.42 ± 0.05b	0.0578 ± 0.0068cd	489.01 ± 39a	99.65
		Ry	43, 318 ± 1, 204	345 ± 3b	3.65 ± 0.04a	0.0542 ± 0.0084d	419.32 ± 6bc	99.68
		C	44, 516 ± 34	324 ± 3c	3.46 ± 0.12b	0.0689 ± 0.0016bc	422.98 ± 1b	99.66
		Ra	37, 513 ± 534	353 ± 6b	3.26 ± 0.01c	0.0755 ± 0.0126b	411.55 ± 5bc	99.67
		M	32, 617 ± 7	309 ± 10cd	3.27 ± 0.10c	0.0762 ± 0.0023b	392.08 ± 7c	99.70
		CM	41, 255 ± 4, 568	292 ± 10d	2.94 ± 0.00d	0.1218 ± 0.0052a	429.26 ± 3b	99.68

*Data with different letters in each column indicate significantly different between treatments at 0.05 level*.

**Figure 2:**
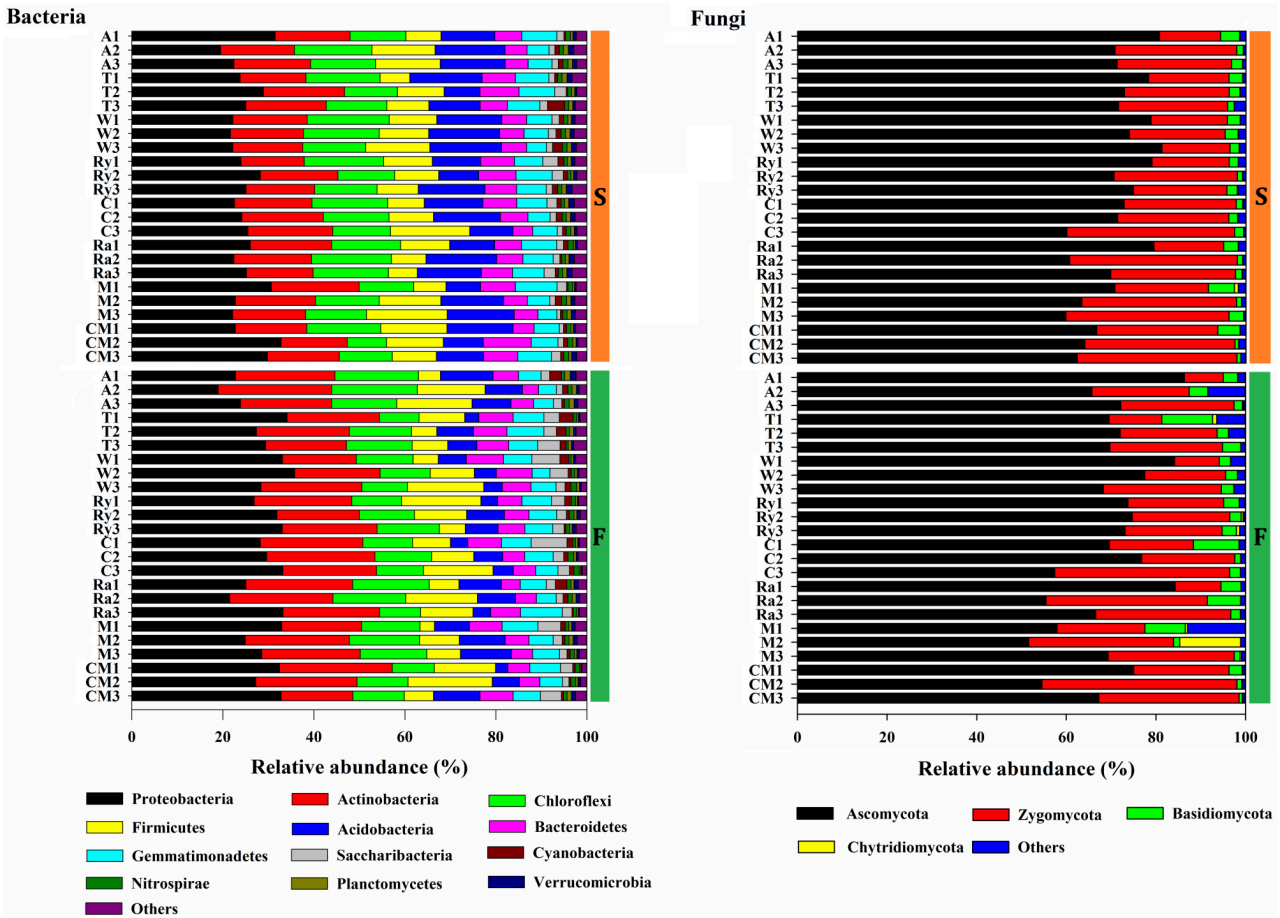
Changes in the relative abundances of bacterial and fungal phyla under different treatments in the spring (S) and fall (F) cropping seasons. Others includes phyla below 0.1% of relative abundance and the unclassified phyla.

**Figure 3:**
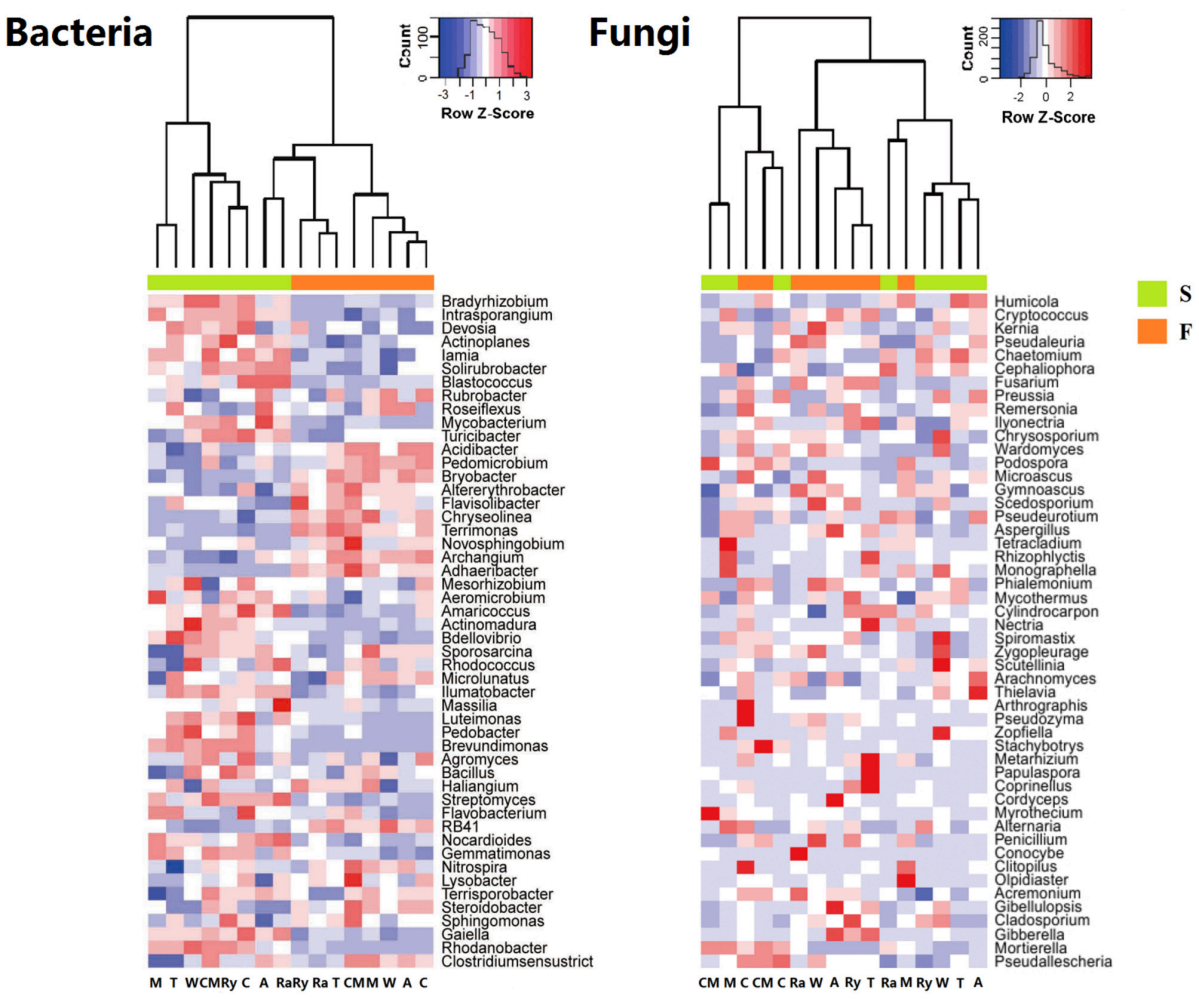
Heatmap of top 50 genera of soil bacterial and fungal communities in the spring (S) and fall (F) cropping seasons. Legends showed the Z-scores, demonstrating all samples were represented by the median-centered Z-scores as the relative abundance levels.

The predominant phyla of bacterial community were *Proteobacteria, Actinobacteria, Chloroflexi, Firmicutes, Acidobacteria, Bacteroidetes*, and *Gemmatimonadetes*, these phyla occupied more than 90% of the total sequences (Figure 2). In addition, *Saccharibacteria, Cyanobacteria, Planctomycetes, Verrucomicrobia, Nitrospirae, Latescibacteria*, and *Parcubacteria* were detected at relatively low abundances (relative abundance < 1%). *Ascomycota, Zygomycota*, and *Basidiomycota* were the dominant fungal phyla at all treatments, which accounted for more than 95% of the sequences, and *Chytridiomycota* was minor phyla, with a relatively lower abundance (Figure 2). Figure 3 demonstrated that the influence of intercropping systems on the 50 most abundant genera of soil bacterial and fungal communities in the two growing seasons. Relative abundance of soil microbial community from high to low is represented by red through white to blue. Of bacterial community, intercropping systems increased relative abundances of *Aeromicrobium* and *Nocardioides*, but *Bradyrhizobium, Clostridium_sensu_stricto_1, Acidibacter, IIumatobacter*, and *Sterodobacter* of intercropping systems had relative low abundances in the two growing seasons (Figure 3). With regard to soil fungal genera, *Chaetomium, Gymnoascus*, and *Arthrographis* had relative high abundances in the intercropping systems, and *Mortierella* was just the opposite, compared with monoculture in the two growing seasons (Figure 3).

### Soil bacterial and fungal communities structure

**Figure 4:**
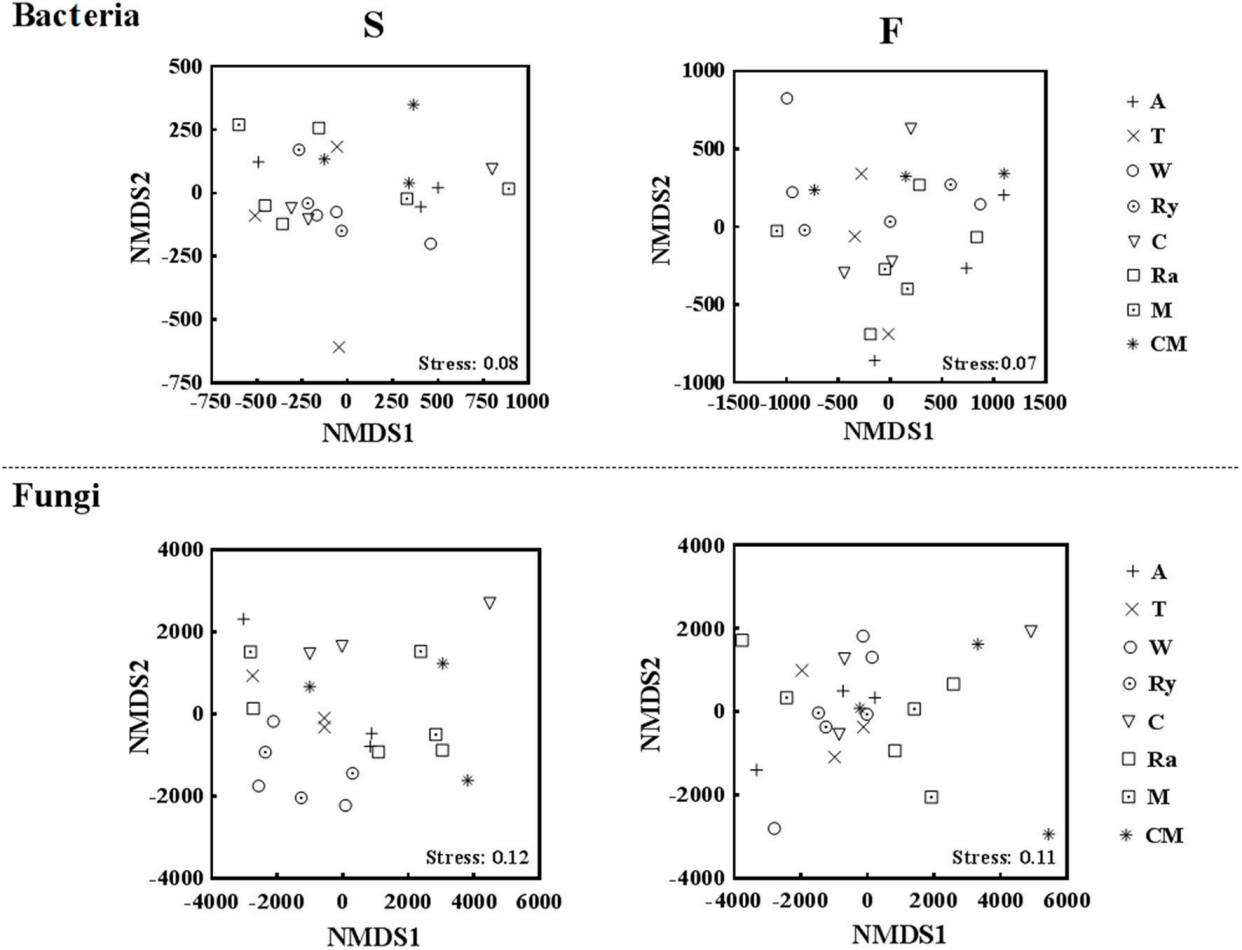
Nonmetric multidimensional scaling (NMDS) based on euclidean distance plot of all soil bacterial and fungal communities in the spring (S) and fall (F) cropping seasons.

NMDS analysis at the OTU level showed that the differences of bacterial and fungal β-diversity based on the euclidean distance dissimilarity (Figure 4). The NMDS plot illustrated that triplicates of the same treatment were not situated closely, and soil bacterial and fungal communities of all samples had a distinct difference in the two growing seasons (Table 3). In addition, there was no obvious distinction between intercropping systems and monoculture in spring, but mustard-cucumber and alfalfa-cucumber systems were separated from monoculture in fall (Figure 4). For soil fungal community, alfalfa-cucumber, trifolium-cucumber, wheat-cucumber and rye-cucumber systems had a relatively distinguishing boundaries with monoculture in the two growing seasons (Figure 4).

**Table 3:** Dissimilarity comparison of soil microbial communities structure between spring and fall cropping seasons.

**Spring vs. Fall**	**ANOSIM**		**Adonis**			**MRPP**		
	***R***	***P***	***F***	***R*^2^**	***P***	**Delta (δ)**	**Effect size (A)**	***P***
Bacaterial community	0.666	0.001	14.13	0.235	0.001	0.354	0.112	0.001
Fungal community	0.197	0.001	5.088	0.100	0.001	0.353	0.043	0.001

**Figure 5:**
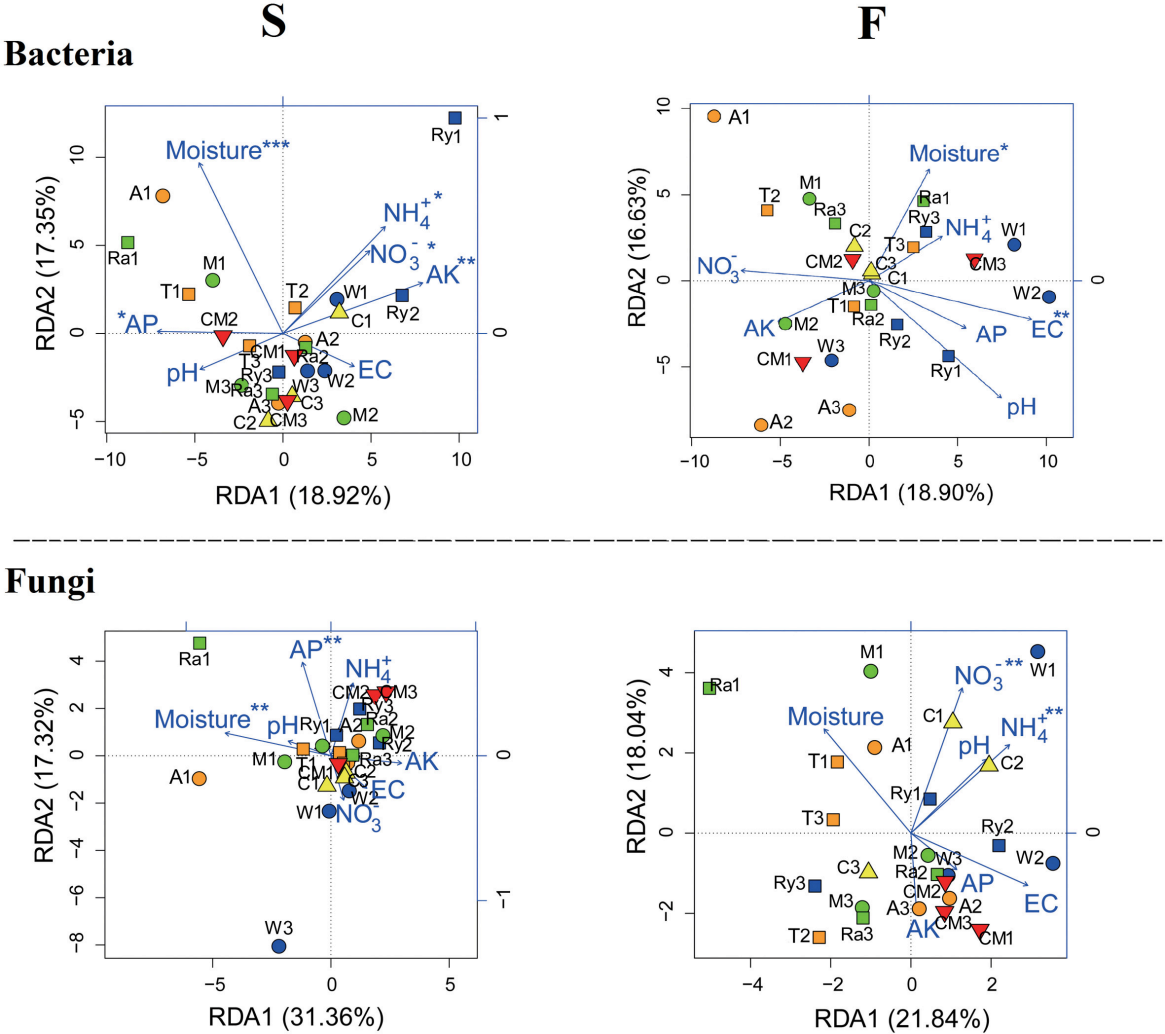
Redundancy analysis (RDA) demonstrating the relationships between soil environmental factors and soil microbial communities during spring (S) and fall (F) cropping seasons in the control (CM), alfalfa (A), trifolium (T), wheat (W), chrysanthemum (C), rye (Ry), mustard (M), and rape (Ra). *P < 0.05, **P < 0.01, ***P < 0.001.

Redundancy analysis (RDA) (Figure 5) used to shed light on the influence of variation of soil physicochemical characteristics on the soil microbial community structure and compositions was showed that soil moisture and AP were the crucial environmental variations that correlated with the soil bacterial and fungal communities composition among soil samples in spring. In fall, EC and soil moisture were strongly correlated with soil bacterial community, however, soil fungal community was mainly influenced by soil ${\text{NO}}_{3}^{-}$-N and ${\text{NH}}_{4}^{+}$-N.

### Co-occurrence network analysis

**Figure 6:**
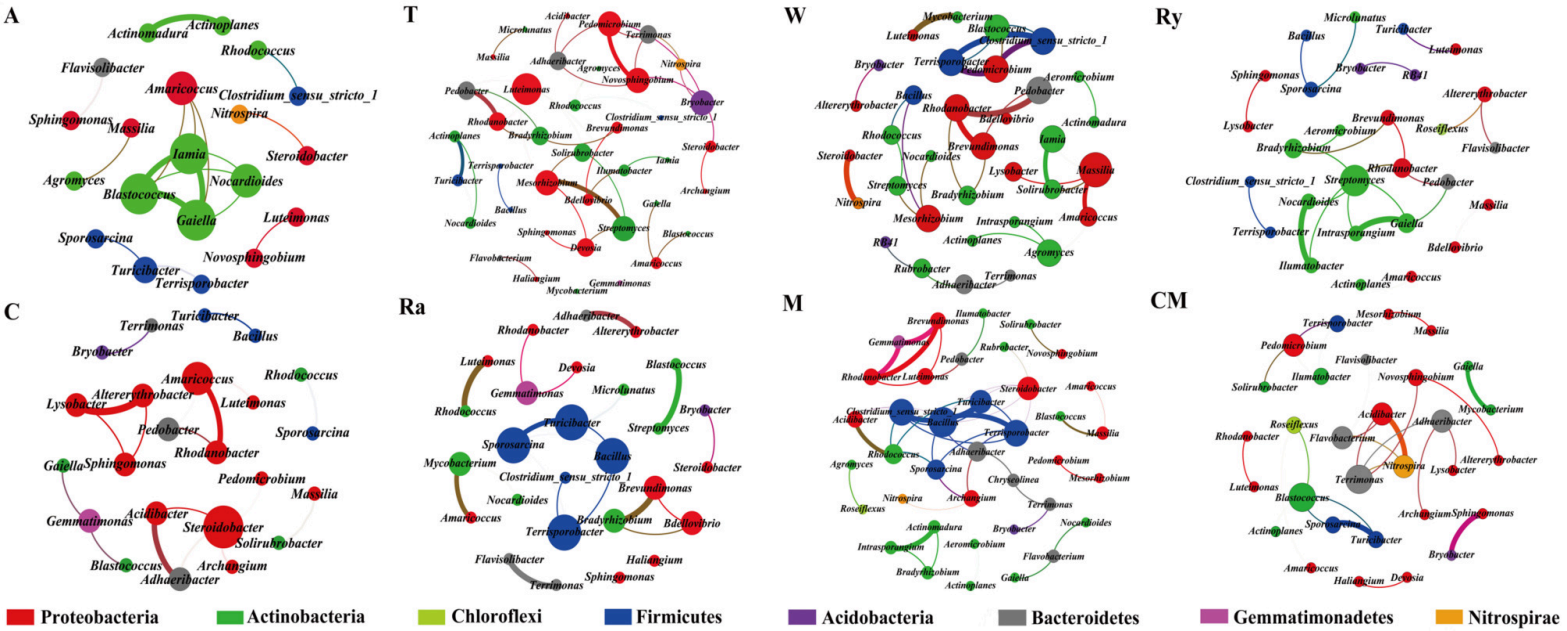
Network of co-occurring bacterial genera based on correlation analysis for intercropping cultivations and cucumber monoculture. A connection stands for a strong (Spearman's ρ > 0.6) and significant (*P* < 0.01) correlation. The size of each node is proportional to the degree, the thickness of each edge is proportional to the value of Spearman's correlation coefficients. Nodes colored by taxonomy.

**Figure 7:**
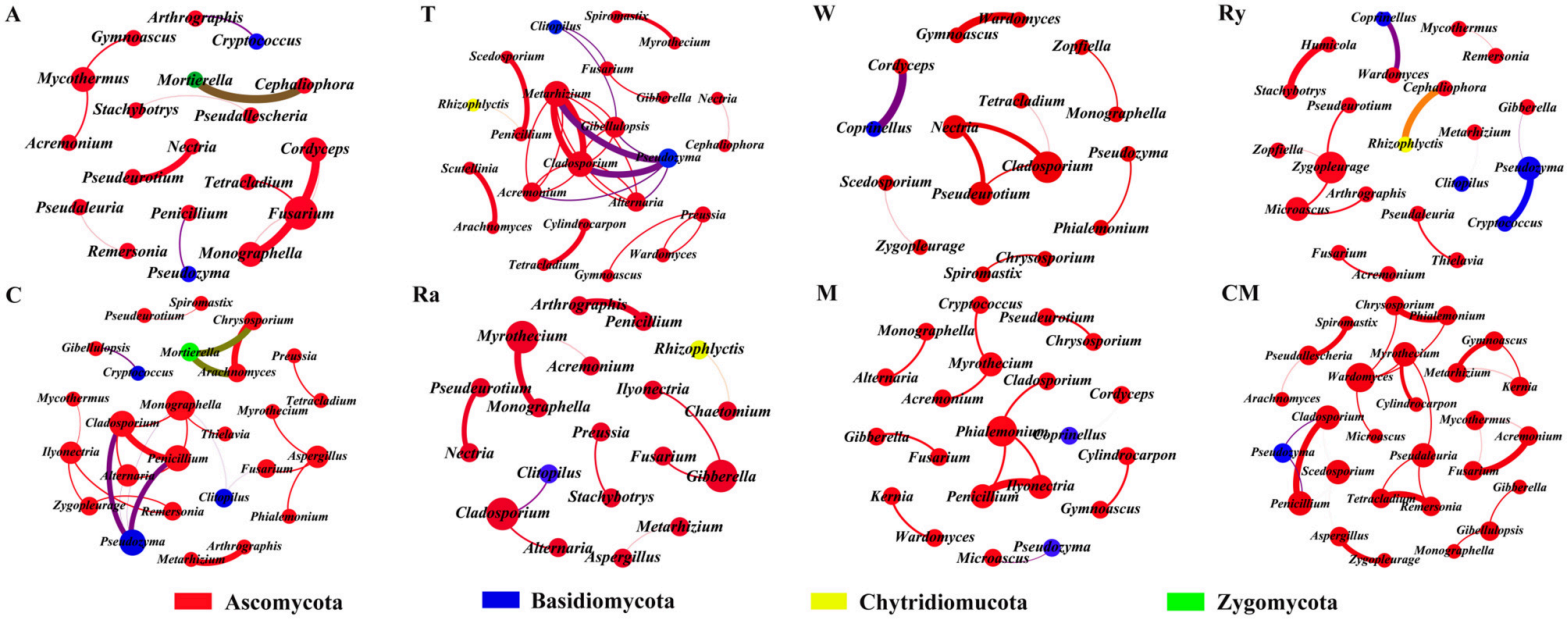
Network of co-occurring fungal genera based on correlations analysis for intercropping cultivations and cucumber monoculture. A connection stands for a strong (Spearman's ρ > 0.6) and significant (*P* < 0.01) correlation. The size of each node is proportional to the degree, the thickness of each edge is proportional to the value of Spearman's correlation coefficients. Nodes colored by taxonomy.

Most of the analytical methods based on metagenomic data focus on single properties of the studied communities, with little attention to the interactions between microbial species. Network analysis is widely performed to explore the interactions of microbial taxon in the complex microbial communities. In this study, network analysis was applied to illustrate the differences of soil bacterial and fungal communities between intercropping systems and monoculture in the two growing seasons (Figures 6, 7), and the network properties of soil bacterial and fungal communities were summarized in Table 4. The structural features, nodes and edges, of bacterial networks under trifolium-cucumber, mustard-cucumber, and wheat-cucumber systems were higher than under cucumber monoculture, showing that they had more connection and closer relationships of soil microbial taxa. For fungal community, the intercropping systems, especially for wheat-cucumber systems, had a less connection and more alienated relationships, compare with the monoculture. The hubs connecting mostly with members of others in intercropping systems were *Blastococcus, Luteimonas, Massiliu, Streptomyces, Steroidobacter, Sporosarcina*, and *Bacillus*, belonging to the phyla of *Proteobacteria, Actinobacteria*, and *Firmicutes*, and *Terrimonas*, the phylum of *Bacteroidetes*, was the major taxa in the monoculture (Figure 6). Of fungal networks, The keystone species of intercropping systems were *Fusarium, Cladosporium, Zygopleurage, Monographella, Myrothecium, Gibberella*, and *Phialemonium*; *Wardomyces* was hub connected chiefly with other species under the monoculture cultivation (Figure 7).

**Table 4:** Network properties of soil bacterial and fungal communities.

**Classified**	**Treatments**	**Average degree (AD)**	**Network diameter (ND)**	**Average path length (APL)**	**Clustering coefficient (CC)**	**Modularity (MD)**	**Nodes**	**Edges**
Bacteria	A	1.700	2	1.105	0.750	0.682	20	17
	T	2.103	9	2.803	0.416	0.756	39	41
	W	1.941	4	1.797	0.483	0.829	34	33
	Ry	1.643	4	1.842	0.315	0.678	28	23
	C	1.565	2	1.308	0.750	0.821	23	18
	Ra	1.481	4	1.467	0.556	0.823	27	20
	M	2.368	6	2.368	0.448	0.645	38	45
	CM	1.733	5	2.169	0.487	0.762	30	26
Fungi	A	1.263	2	1.200	0.583	0.820	19	12
	T	2.696	5	1.978	0.460	0.691	23	31
	W	1.250	2	1.167	0.778	0.781	16	10
	Ry	1.182	3	1.450	0.482	0.842	22	13
	C	2.000	6	2.471	0.652	0.727	26	26
	Ra	1.158	2	1.214	0.576	0.860	19	11
	M	1.238	2	1.188	0.583	0.838	21	13
	CM	2.000	4	2.000	0.658	0.786	27	27

## Discussion

Intercropping is thought to be an environmentally friendly method for disease management and nutrient complementary, and could also change the microclimatic conditions (Li et al., 1999, 2014; Cong et al., 2015). Previous papers showed that soil physicochemical properties, soil carbon and nitrogen, soil bulk density and pH, are improved by intercropping systems (Morris and Garrity, 1993; Fan et al., 2006; Bedoussac and Justes, 2009; Liu et al., 2014). We found that intercropping systems did not significantly change the continuous cropping soil physicochemical characteristics such as soil moisture, pH, EC, and AK in the two growing seasons (Table 1). These results were likely caused by short-term test and the complex environment of greenhouse. However, soil AP content was significantly decreased under intercropping systems, which may be caused by the competitive interaction among neighboring plants (Guadet and Keddy, 1995; Goldberg, 1996; Zhang and Lamb, 2011).

According to the qPCR results, wheat-, trifolium-, and mustard-cucumber systems increased the continuous cropping soil bacterial absolute abundance, and fungal abundance was increased under rye-cucumber systems, but decreased under wheat- and rape-cucumber systems in the two growing seasons (Figure 1). The results were in line with the previous findings that intercropping changed soil microbial abundance (Tjamos et al., 1992; Zhou et al., 2011). Soil microbial diversity is closely related to soil ecosystem stability and nutrient transformation, and intercropping could manage to various agroecosystem services by improving soil quality (Cong et al., 2015). Our results showed that soil microbial diversity indices were increased under intercropping systems (Table 2), indicating that intercropping improved continuous cropping soil quality and contribute to strengthen the stability of facility ecosystem.

Plant species, root exudates and soil types could affect soil microbial community (Marschner et al., 2001; Wieland et al., 2001; Broeckling et al., 2008; Lauber et al., 2008, 2009). Soil microbial taxonomic composition forcefully changed between intercropping and cucumber continuous cropping soils (Figures 2, 3). *Proteobacteria* was the most abundant phylum in the cucumber continuous cropping soil, but *Actinobacteria* and *Chloroflexi* were more abundant under the intercropping systems. This finding is in line with a previous study that *Actinobacteria* and *Chloroflexi* were present at a higher percentage in mulberry-soybean intercropping systems (Li et al., 2016). For fungal community, *Ascomycota* in the wheat-cucumber system was the most abundant phyla compared to other treatments, the result is also in accordance with a previous study in which *Ascomycota* were the main phyla in the wheat monoculture and intercropping systems (Granzow et al., 2017). Additionally, in this study we found that genera *Aeromicrobium, Nocardioides* and *Gymnoascus* were enriched under intercropping systems, but *Clostridium_sensu_stricto_1, Acidibacter, IIumatobacter, Steroidobacter*, and *Mortierella* were inverse. *Aeromicrobium* and *Nocardioides* belong to *Actinobacteria, Actinobacteria* were well-known for species that have beneficial association with plants (Kim et al., 2011; Palaniyandi et al., 2013). *Mortierella* could generate antagonistic substance, Arachidonic acid, which is an elicitor of phytoalexins in plants to suppress plant disease (Eroshin et al., 1996; Tagawa et al., 2010). It was decreased in the intercropping treatments, the soil is a complex environment, the concrete reasons need to be further studied in the future. High concentrations of soil available phosphorous and host species determined the arbuscular mycorrhiza (AM) community, that is the reason why *Glomeromycota*, concluded AM that can improve the nutrients supply and soil structure, was not detected in this experiments (Parniske, 2008; Gosling et al., 2013). Many studies indicated that *Bradyrhizobium* not only have a wide range of legumes, but also are abundant in non-legume plants (Trinick and Hadobas, 1988; VanInsberghe et al., 2015). We found that wheat-cucumber and mustard-cucumber intercropping systems had a higher relative abundances of *Bradyrihizobium* in spring and fall, respectively.

NMDS and RDA analyses demonstrated that soil microbial community structure did not have significant changes between intercropping and monoculture soils (Figures 4, 5). However, bacterial and fungal communities structure were significantly altered in the two growing seasons (Table 3). Previous studies showed that temperature regulated strongly soil microbial community structure and soil microbial communities varied broadly in their suitable temperature (Alster et al., 2016). Temperature of two growing seasons had a great difference in the northeast of China, thus, we speculate that the key factor was temperature led to variations of soil microbial communities structure in the two growing seasons. Soil moisture was related to soil microbial distribution, Banerjee et al. (2016) reported that soil moisture play an important role in soil microbial activities and composition. We found that soil moisture, AP, EC, ${\text{NH}}_{4}^{+}$-N, and ${\text{NO}}_{3}^{-}$-N were related to soil microbial community structure (Figure 5). These results illustrated that soil characteristics indirectly influenced soil bacterial and fungal community structure. In our study, intercropping did not significantly affect soil microbial community structure. These results need to carry out long-term test to confirm in the future.

A network analysis was performed to compare the complexity of operating in the intercropping soil and the continuous cropping soil. Network analysis was constructed using all positive correlations of top 50 most abundant genera of soil microbial community (Figure 6). The number of correlations of fungal community was lower in the intercropping systems than in the control, and bacterial community of trifolium, mustard, and wheat intercropping systems, especially wheat intercropping systems, had more positive correlations than the control (Table 4). In previous study, researchers found that rhizosphere of wild oat had the more complex networks compared to the bulk soils, it means that rhizosphere is contributed to the interaction between soil microbial taxon (Shi et al., 2016). In addition, plants have the same great effect on soil microbial communities as soil, intercropping systems could increase plant diversity, different plant species secrete diverse root exudates, which can affect species-specific shift in the microbial community as well as the soil microbial diversity (Grayston et al., 1998; Marchner et al., 2004; Broeckling et al., 2008; Zhou and Wu, 2012; Li et al., 2014). Plant residues can regulate the soil biochemical cycle, so that it generate strong microbial activities and interactions, and the amount and degradation rate of plant residues have great differences among intercropping treatments, which may also be the reason for the differences in the microbes interactions of all treatments (Martens, 2000; Philippot et al., 2013). Moreover, Soil microbial interactions can create intense positive feedbacks in the plant communities, which might contribute directly to decrease plant diversity (Bever et al., 2010). Now that we have determined the differences between intercropping and continuous cropping soils with respect to soil microbial communities and soil physicochemical properties, this will provide theoretical basis for microbial remediation of continuous cropping soil in the future.

## Conclusions

In summary, our results illustrated that trifolium-cucumber and mustard-cucumber systems increased remarkably the bacterial diversity, and fungal diversity was more abundant in the intercropping systems. However, soil microbial community structure was not significantly altered by seven intercropping crops. We found that soil microbial community varied with soil characteristics, indicating the effect of intercropping on soil microbial community was indirectly affected by soil environmental factors. Moreover, the bacterial and fungal co-occurrence patterns were influenced by intercropping systems in essence. Intercropping systems had a complex relationships between soil bacterial community, and had less connection and relationships of fungal taxa.

## Author contributions

FW designed this experiment. SL executed the experiment and finished the manuscript.

### Conflict of interest statement

The authors declare that the research was conducted in the absence of any commercial or financial relationships that could be construed as a potential conflict of interest.
